# Transcriptional Regulation of *Culex pipiens* Mosquitoes by *Wolbachia* Influences Cytoplasmic Incompatibility

**DOI:** 10.1371/journal.ppat.1003647

**Published:** 2013-10-31

**Authors:** Sofia B. Pinto, Kirsty Stainton, Simon Harris, Zakaria Kambris, Elizabeth R. Sutton, Michael B. Bonsall, Julian Parkhill, Steven P. Sinkins

**Affiliations:** 1 Peter Medawar Building for Pathogen Research and Nuffield Department of Medicine (NDM), University of Oxford, Oxford, United Kingdom; 2 Department of Zoology, University of Oxford, Oxford, United Kingdom; 3 Pathogen Genomics, Wellcome Trust Sanger Institute, Wellcome Trust Genome Campus, Hinxton, Cambridge, United Kingdom; Institut Pasteur, France

## Abstract

Cytoplasmic incompatibility (CI) induced by the endosymbiont *Wolbachia pipientis* causes complex patterns of crossing sterility between populations of the *Culex pipiens* group of mosquitoes. The molecular basis of the phenotype is yet to be defined. In order to investigate what host changes may underlie CI at the molecular level, we examined the transcription of a homolog of the Drosophila melanogaster gene grauzone that encodes a zinc finger protein and acts as a regulator of female meiosis, in which mutations can cause sterility. Upregulation was observed in Wolbachia-infected C. pipiens group individuals relative to *Wolbachia*-cured lines and the level of upregulation differed between lines that were reproductively incompatible. Knockdown analysis of this gene using RNAi showed an effect on hatch rates in a Wolbachia infected Culex molestus line. Furthermore, in later stages of development an effect on developmental progression in CI embryos occurs in bidirectionally incompatible crosses. The genome of a *w*Pip *Wolbachia* strain variant from *Culex molestus* was sequenced and compared with the genome of a *w*Pip variant with which it was incompatible. Three genes in inserted or deleted regions were newly identified in the *C. molestus w*Pip genome, one of which is a transcriptional regulator labelled *wtrM*. When this gene was transfected into adult *Culex* mosquitoes, upregulation of the *grauzone* homolog was observed. These data suggest that Wolbachia-mediated regulation of host gene expression is a component of the mechanism of cytoplasmic incompatibility.

## Author Summary

*Wolbachia* are maternally inherited bacteria that manipulate invertebrate reproduction. Cytoplasmic incompatibility is embryo death that occurs when males carrying *Wolbachia* mate with females that do not, or that carry a different *Wolbachia* variant; its mechanism is poorly understood. In *Culex* mosquitoes, in the presence of *Wolbachia* a gene related to a *Drosophila melanogaster* gene, *grauzone*, which has been shown to act as a regulator of the meiotic cell cycle, showed an elevated level of expression. When lower levels of expression were achieved through RNA interference, embryo hatch rates were affected and the stage of development at which embryo death occurs was altered. To find *Wolbachia* genes that influence cytoplasmic incompatibility, we compared the genomes of two variants of *Wolbachia* from *Culex* that produce cytoplasmic incompatibility with one another. Although most segments of these genomes were very similar, one newly identified gene is predicted to be a regulator of gene transcription. We cloned this gene into a plasmid, expressed it in adult mosquitoes and found higher levels of expression of the *Culex grauzone* homolog. This suggests that the *Wolbachia* transcriptional regulator may play an important role in manipulating the host in order to induce cytoplasmic incompatibility.

## Introduction

The intracellular maternally inherited bacterium Wolbachia pipientis, a widespread endosymbiont of invertebrates [1], can influence reproduction in arthropods. The most common manipulation is cytoplasmic incompatibility (CI). Sperm from Wolbachia-infected males are modified during maturation, prior to the loss of Wolbachia, such that aberrant events in the male pronucleus [2]–[7] lead to embryo developmental arrest when these sperm fertilize eggs from uninfected females. However, progeny are rescued when both parents carry compatible Wolbachia and therefore, infected females have a selective advantage under this unidirectional pattern of CI.

The Culex pipiens group of sibling species of mosquito, in which CI was first discovered, provides a model system with useful features for examining the genetic differences that underlie CI. Even though only one designated strain of *Wolbachia* (*w*Pip) is present in *Culex pipiens*, complex crossing types including both unidirectional and bidirectional CI [8]–[14] occur between populations. Compatibility or partial CI is most often observed, but certain lines will be completely incompatible in one or both crossing directions with a majority of other *C. pipiens* lines. Understanding the basis of this complexity has been a long standing problem.

Several studies have shown that Wolbachia and its products can influence host gene transcription, notably a major upregulation of immune genes in transinfected naïve mosquitoes [15]–[21], which can contribute to the inhibition of arboviruses and Plasmodium parasites [18], [20], [22]. Differential regulation of several candidate genes has also been observed in Drosophila [23]–[25]; these genes have been hypothesized to form part of the CI phenotype, but so far it has not been possible to confirm a role in incompatibility generation.

Cytological studies in Drosophila and Nasonia have revealed that aberrant events in the male pronucleus in an incompatible fertilization include abnormal histone H3.3 and H4 deposition, prolonged or incomplete DNA replication, delayed chromosome condensation/segregation and nuclear envelope breakdown [2]–[7]. It has been suggested that cell cycle defects in the male pronucleus in CI embryos could be due to disruption of cell cycle regulators or the induction of checkpoints that control entry into mitosis, possibly at the metaphase to anaphase transition [5], [6]; these defects are rescued by Wolbachia in females. The D. melanogaster gene grauzone (grau) encodes a zinc finger transcription factor that plays a role in the regulation of the female meiotic cell cycle [26], [27]. Meiotic arrest at metaphase I is released by egg activation, resulting in completion of the two meiotic divisions; mothers mutant for grau are sterile and lay eggs presenting aberrant chromosomal segregations at meiosis I, which arrest their development in metaphase of meiosis II [26]. Among the many cell cycle regulating genes that have been described, *grau* is one of only two genes known to be involved in regulation of metaphase II. Given the parallels between abnormal chromosome segregations in both CI embryonic arrest and in *grau* mutant embryos, and the similarities between metaphase-anaphase transition in meiosis II and mitosis, we considered this gene a candidate for involvement in CI generation.

Not much is known about which *Wolbachia* genes are responsible for the manipulation of host early embryogenesis. The lack of a transformation system for this intracellular symbiont, which cannot be cloned or cultured outside of host cells, has meant that systematic testing of the phenotypic effects of candidate genes has not been possible. The Pel line of *Culex quinquefasciatus* from which a *w*Pip genome sequence was generated [28] has been shown to be bidirectionally incompatible with a *Culex molestus* line [29]; comparative genome analyses between these incompatible *Wolbachia* strains could therefore be used to determine differences between them that may be involved in the generation of different crossing types. Comparisons with the JHB line *w*Pip genome [30], [31] are also possible. Therefore, the aims of this study were to identify a homolog of grau in *Culex* and test its potential involvement in the mechanism of CI, and in parallel to purify, sequence and analyse the *w*PipMol genome in order to attempt to identify *Wolbachia* genes that may be involved in manipulation of the host, particularly in transcriptional modification.

## Results

### Transcriptional analysis of *CPIJ005623*

We identified homologs of the D. melanogaster gene grau in Culex quinquefasciatus (a member of the C. pipiens complex) by interrogation of the genome database [31], which revealed the presence of two paralogs: CPIJ005623 and CPIJ015950. These two paralogs are identical with the exception of a 56 nucleotide (nt) indel between nt 994-1049 of CPIJ005623 which is absent in CPIJ015950, one nt change leading to one amino acid substitution and one nt deletion near the exon1-exon2 border of CPIJ015950 leading to two amino acid substitutions. PCR analysis revealed that only CPIJ005623 was present in the Culex lines used in this study (Figure S1), suggesting that they may just be allelic variants.

**Figure 1:**
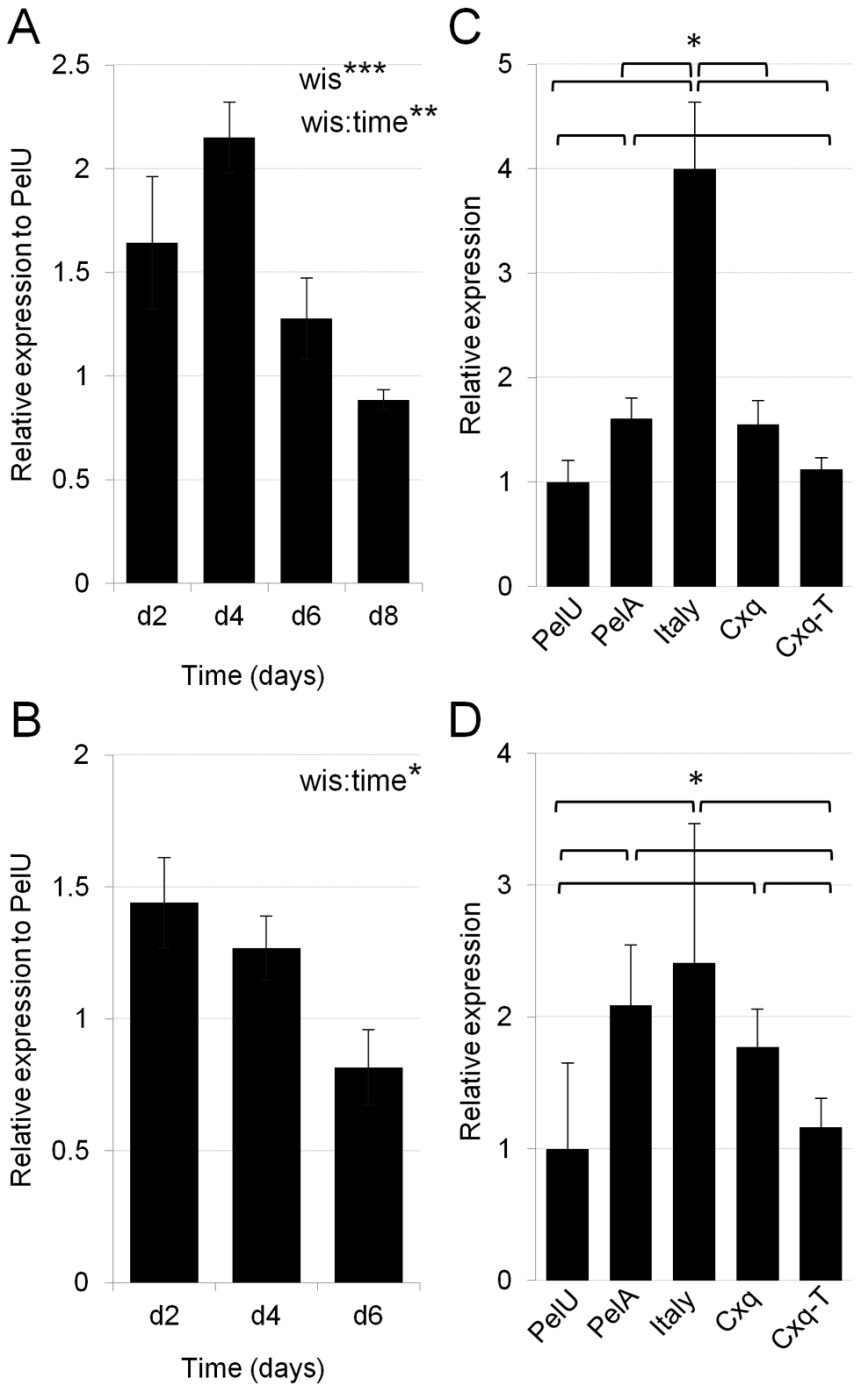
Transcription analysis of *CPIJ005623* in the *Culex pipiens* complex. **A–B**. *CPIJ005623* transcription in adult female (A) and male (B) *Culex quinquefasciatus*, Pel (*Wolbachia*-infected) line relative to the Wolbachia-uninfected PelU, over time (days post pupal eclosion). Similar decreasing expression dynamics was seen in both sexes. **C–D**. Tissue analysis of *CPIJ005623* transcription at 4 d in ovaries (C) and 1 d in testes (D) in *C. quinquefasciatus Wolbachia*-infected lines Pel and Cxq (*30*), their *Wolbachia*-free counterparts PelU and CxqT, and *Wolbachia*-infected Italy line *C. molestus*. Upregulation of *CPIJ005623* expression is seen in all *Wolbachia*-infected lines. Average of the mean values of four biological repeats (+/− standard error-SE) are presented. Two-way ANOVA statistical analysis was used to determine effect of *Wolbachia* infection status (wis) and time on *CPIJ005623* expression. Wilcoxon rank-sum test was used to determine differences between *Wolbachia* infection status in *CPIJ005623* expression (C–D): *p<0.05, **p<0.01, ***p<0.001.

Relative transcription of CPIJ005623 was measured in members of the C. pipiens complex using quantitative reverse transcription PCR (qRT-PCR). We first looked at the transcription of the gene in whole adults over time to understand its expression dynamics in individual mosquitoes that carry Wolbachia and their antibiotic-treated Wolbachia-free genetic counterparts. In Wolbachia-infected C. quinquefasciatus Pel adult females, CPIJ005623 transcription showed a peak of over two-fold upregulation compared to the Wolbachia-cured line PelU at 4 days post pupal eclosion (dpe), which then decreased to similar levels as the PelU line by 8 dpe (Figure 1A). Differences in CPIJ005623 transcript levels were found, with an interaction between Wolbachia infection status (wis) and developmental time (Two-way Anova: wis:time- F = 7.66, Df = 1, p<0.01), where the difference between wis contributes most strongly to the interaction (Two-way Anova: wis- F = 13.53, Df = 1, p<0.001). In males (Figure 1B), there was an interaction between Wolbachia infection status (wis) and developmental time (Two-way Anova: wis:time- F = 5.79, Df = 1, p = 0.025) which was independent from any difference seen at 6 dpe between the Wolbachia-infected and uninfected males (Welch's t-test: t = 0.78, Df = 4.833, p = 0.4716).

We then focused our analysis at single time points where the expression was found to be highest, separately in ovaries (4 dpe) and testes (2 dpe) (the tissues where CI is expressed). A similar pattern of upregulation (Wilcoxon rank-sum: p<0.05) of CPIJ005623 in Wolbachia-infected ovaries (Figure 1C) and testes (Figure 1D) was seen for two different pairs of C. quinquefasciatus infected (Pel and Cxq) versus cured (PelU and CxqT) lines, and for an infected Culex molestus line. A higher level of upregulation in C. molestus Italy ovaries was observed than in Pel females.

### Knockdown of *CPIJ005623* in *C. molestus* females

**Figure 2:**
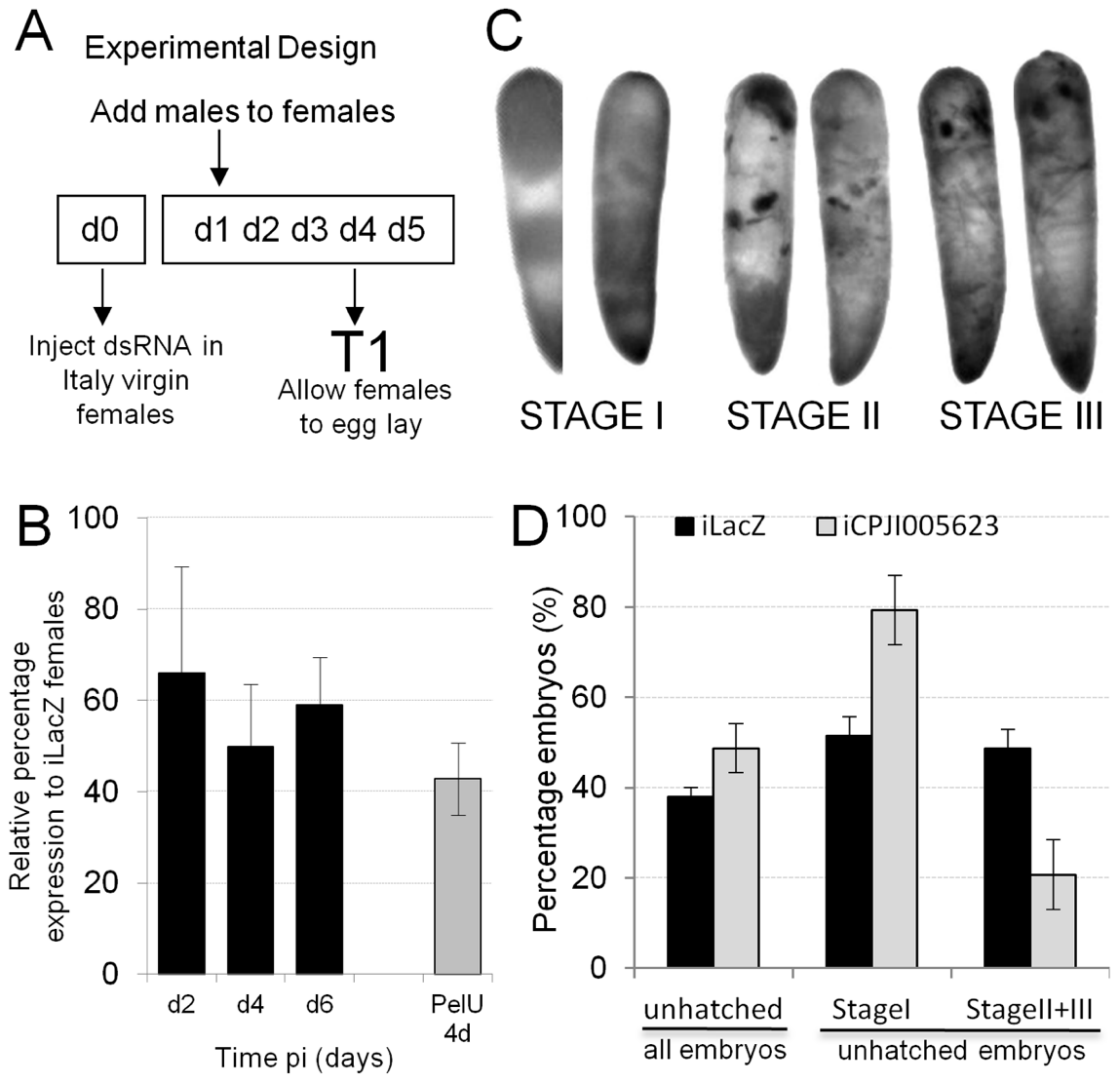
Knockdown analysis of *CPIJ005623* in *C. molestus* Italy females. **A**. Diagram representing experimental design of knockdown (KD) experiments, d-day post pupal eclosion, T-time point. **B**. KD assessment of i*CPIJ005623* in Italy ovaries. Reduction of *CPIJ005623* mRNA levels similar to uninfected ovaries was observed in infected ovaries when CI developmental progression was seen. **C**. Picture examples of developmental progression in *Culex* embryos undergoing CI, Stage I: early arrest, Stage II and III: late arrest. **D**. Increased percentage of unhatched embryos mimic a reduced rescue function. Also increased early embryo (stageI) developmental arrest is detected after i*CPIJ005623* in compatible Italy male cross.

Given the upregulation of CPIJ005623 detected in the Italy Wolbachia-infected line compared to uninfected females, we knocked down the gene in infected females using dsRNA to investigate whether this change may be involved in the rescue function of CI. Restoring transcription to uninfected levels in these females might result in developmental arrest in embryos from crosses with Wolbachia-infected males (Figure 2A). Knockdown (KD) levels were assessed in dissected ovaries and a reduction of approximately 50% was detected in the transcription of CPIJ005623 in iCPIJ005623-injected females compared to iLacZ-injected control-KD females at 4 days post injection (dpi) which were similar to PelU ovarian expression levels (Figure 2B). An increase in the number of unhatched eggs was detected in a compatible cross of Italy males with iCPIJ005623 females (48.7%) compared to control-KDs (37.9%) (Table 1- Welch's t-test: t = 8.975, Df = 39.861, p<0.0001).

**Table 1:** *CPIJ005623* KD effects in *C. molestus* Italy and *C. quinquefasciatus* Pel females in compatible crosses.

Time Point	Gene KD	Ind#	Total egg#	P [%]		*p*-valueφ	Mean embryo/Ind			[%] StI∶III	*p-*valueξ
				H	U		T	STI	STIII		
**Compatible Cross: Italy KD females** × **Italy males**											
T1	i*LacZ*	20	374	62	38		7.1	3.6	3.4	50∶50	
	i*CPJI005623*	20	396	51	49	***<0.0001***	**9.6**	**7.6**	**2**	**80∶20**	***<0.0001***
**Compatible Cross: Pel KD females** × **Pel males**											
T1	i*LacZ*	19	1450	88	12		9	3	6	30∶70	
	i*CPJI005623*	19	1235	83	16	0.588	11	7	4	**60∶40**	***<0.0001***

KD, Knockdown; Ind #, number of Individuals, P[%], percentage of hatched (H) and unhatched (U) embryos;

φ *p-*value, Welch's t-test on difference between hatched and unhatched embryos;

Mean embryo/ind, mean number of unhatched embryos undergoing CI and scored for developmental progression stage per individual; T = total; STI, stage I; STIII, stage II and III;

ξ *p-*value on difference between stage I and stage II&III embryo proportions determined via GLM with binomial error structures.

In Culex and other Diptera, arrest can occur at an early (stage I) or late (stage II+III) stage in embryo development [3], [12], [32] and these stages are identified by distinctive embryo morphologies (Figure 2C). The proportions of stage I and stage II/III embryos that did not hatch in the compatible cross after iCPIJ005623 and control-KD differed (GLM: χ^2^ = 28.957, Df = 1, p<0.0001), with a higher proportion of embryos arresting at stage I in iCPIJ005623 females (Figure 2D).

**Figure 3:**
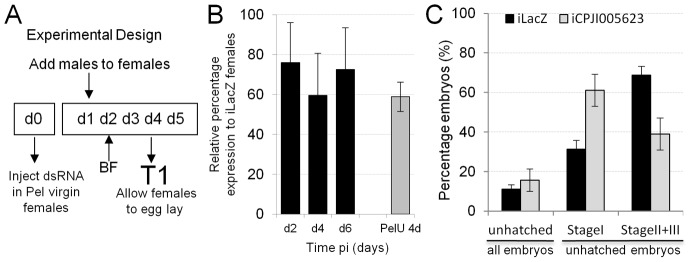
Knockdown analysis of *CPIJ005623* in *C. quinquefasciatus* Pel females. **A**. Diagram representing experimental design of knockdown (KD) experiments, d-day post pupal eclosion, BF-Blood feed, T-time point. **B**. KD assessment of i*CPIJ005623* in Pel ovaries. Reduction of *CPIJ005623* mRNA levels similar to uninfected ovaries was observed in infected ovaries when CI developmental progression was seen. **C**. Increased early embryo (stageI) developmental arrest is detected after i*CPIJ005623* in compatible Pel male cross.

A lower level of upregulation was observed for CPIJ005623 in Pel Wolbachia-infected versus uninfected females (Figure 1B). To understand if grau upregulation is part of a generalized Culex pipiens group rescue response, we performed KD analysis followed by a compatible cross in C. quinquefasciantus Pel females (Figure 3A). Knockdown levels were assessed in dissected ovaries and a reduction of up to 40%, similar to uninfected levels was detected in the transcription of CPIJ005623 compared to control-KDs at 4 days post injection (dpi) (Figure 3B). A higher proportion of embryos arresting at stage I for iCPIJ005623 females was detected than for control-KD females (Figure 3C, Table 1) (GLM: χ^2^ = 23.841, Df = 1, p<0.0001). Although in Pel females unhatched rates were not affected after grau homolog KD, the shift from late to early embryo mortality is conserved in the two Culex lines.

### Knockdown of *CPIJ005623* in *C. pipiens* complex males

**Figure 4:**
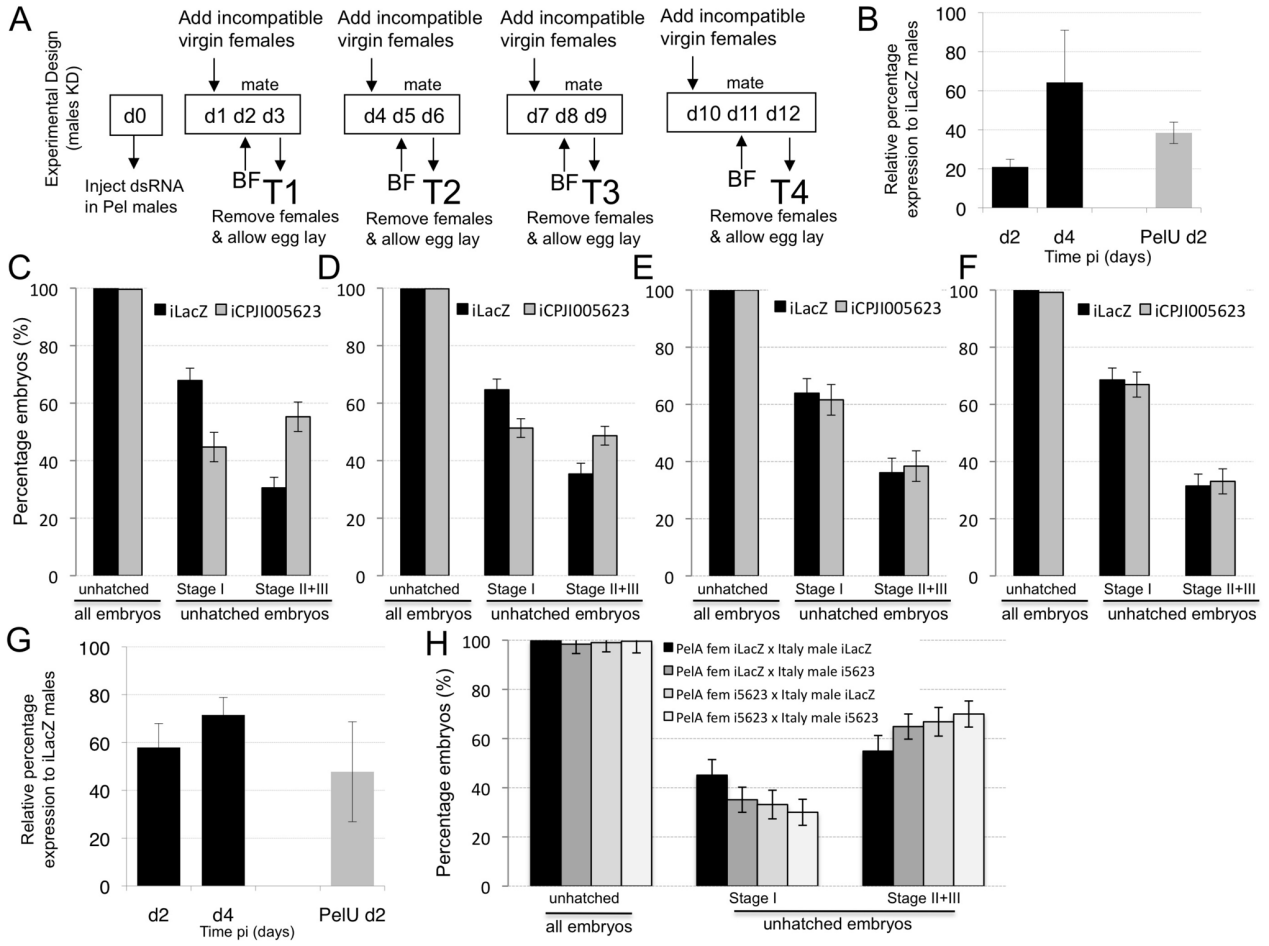
Knockdown analysis of *CPIJ005623* in *C. pipiens* males. **A**. Diagram representing experimental design of knockdown (KD) experiments, d-day post pupal eclosion, BF- Blood feed, T-time point. **B**. KD assessment of i*CPIJ005623* in Pel testis. Reduction of *CPIJ005623* mRNA levels was observed in testis when CI developmental progression was seen. **C–F** Embryo developmental progression after i*CPIJ005623* males in an incompatible Italy female cross. Timepoints under dsRNA early effect (C-T1; D-T2) show an increased proportion of Stage II&III embryos compared to control KDs. No effect was seen at later time points (E-T3; F-T4). **G**. KD assessment of i*CPIJ005623* in Italy testes. **H**. Double KD analysis of *CPIJ005623* in Pel females crossed to Italy males. Increased developmental progression is detected in female, male and double *iCPIJ005623*.

Since CPIJ005623 was also observed to be upregulated in infected versus uninfected males, we tested if this upregulation had a role in inducing CI in crosses between infected males and uninfected females. We failed to obtain an antibiotic-treated Wolbachia uninfected C. molestus line due to the low egg production of this blood meal independent autogenous line. As the upregulation detected was fairly similar and not statistically different between the Wolbachia infected lines tested (Figure 1C) we focused our attention to the C. quinquefasciatus Pel line. The effect of iCPIJ005623 in Pel sperm was assessed over time (Figure 4A), in order to determine if CPIJ005623 transcriptional upregulation produces phenotypic effects either early in spermatogenesis or in mature sperm, given that the average time for spermatogenesis in C. pipiens is 9–11 days [33]. No effect was observed on the survival of embryos produced by Wolbachia-uninfected mothers mated with iCPIJ005623 Wolbachia-infected males. In Culex, when uninfected-females mate with Wolbachia infected-males, most embryos arrest at an early stage and no developmental embryo progression occurs. Knocking down CPIJ005623 in Pel males, by as much as 80% and lower than CPIJ005623 expression levels in the uninfected line (Figure 4B), had no significant effect on developmental progression in unidirectional CI crosses.

The effect of knockdown of CPIJ005623 in an incompatible cross was then examined using C. quinquefasciatus Pel and the C. molestus Italy lines, for which zero egg hatch is observed in both crossing directions. When CPIJ005623 was knocked down in Pel males which were crossed to Italy females, no significant effect on hatch rate was observed, but the percentage of embryos that reached later developmental stages was higher at the two earlier time points tested (GLM: χ^2^ = 65.975, Df = 1, p<0.0001, T1-Figure 4C; χ^2^ = 33.079, Df = 1, p<0.0001, T2-Figure 4D) compared to the progeny of i*L*acZ injected controls (Table 2).

**Table 2:** *CPIJ005623* KD effects in *C. pipiens* males in incompatible crosses.

Time Point	Gene KD	Ind#	Total egg#	Percentage [%]		Mean embryo #/Ind			[%] StI∶II,III	*p-*valueξ
				hatch	unhatch	Total	ST I	ST II&III		
**Incompatible Cross: Italy females** × **Pel KD males**										
T1	i*LacZ*	16	648	0	100	40	26	13	70∶30	
	i*CPJI005623*	16	628	0.37	99.6	39	16	23	**40∶60**	***<0.0001***
T2	i*LacZ*	21	874	0.34	99.7	42	28	14	70∶30	
	i*CPJI005623*	21	941	0.21	99.8	45	23	22	**50∶50**	***<0.0001***
T3	i*LacZ*	15	654	0.15	99.9	44	28	16	70∶30	
	i*CPJI005623*	15	651	0	100	43	27	17	65∶35	0.387
T4	i*LacZ*	16	639	0	100	40	27	13	70∶30	
	i*CPJI005623*	16	632	0.79	99.2	40	26	13	70∶30	0.538
**Incompatible Cross: Pel KD females** × **Italy KD males**										
T1	female iLacZ x male iLacZ	10	399	0	100	40	18	22	45∶55	
	female iLacZ x male i5623	10	373	1.6	98.4	37	13	24	**35∶65**	***0.005***
	female i5623 x male iLacZ	10	410	1	99	41	14	27	**35∶65**	***0.0005***
	female i5623 x male i5623	10	493	0.4	99.6	49	15	34	**30∶70**	***<0.0001***
**Incompatible Cross: Pel KD females** × **Italy males (control)**										
T1	i*LacZ*	32	2594	0.23	99.8	81	36	45	45:55	
	i*CPJI005623*	32	2519	0.16	99.8	79	21	57	**30:70**	***<0.001***

KD, knockdown; Ind #, number of individuals; Mean embryo **#**/ind, mean number of unhatched embryos undergoing CI and scored for developmental progression stage per individual; ST, stage;

ξ p-value on difference between stage I and stage II&III embryo proportions determined via GLM with binomial error structures.

A double knockdown (double-KD) experiment in both Italy males and Pel females was also performed, since upregulation of CPIJ005623 was observed in Pel-infected vs Pel-uninfected ovaries (Figure 1B). A KD of 40% was detected in Italy testes using qRT-PCR analysis (Figure 4G). Crossing results show no significant difference in hatch rates between all four double-KD combinations; however, as before, StageI:StageII/III proportions are significantly different (GLM: χ^2^ = 23.222, Df = 3, p<0.0001) in all crosses where CPIJ005623 transcription was reduced irrespective of the gender of the individual (Figure 4H, Table 2).

### Comparative genomics of *w*Pip

We purified and sequenced the genome of the *w*PipMol strain present in *C. molestus* [29] and together with the previously sequenced *w*PipPel [28] and *w*PipJHB [30], [31] performed a three-way comparative genomic analysis. Crossing experiments revealed that the JHB line of *C. quinquefasciatus* is bidirectionally incompatible with Mol, while JHB and Pel are compatible with each other in both crossing directions (Table S1). Therefore the pattern of genome variation that is initially of most interest, with respect to different crossing types in the *C. pipiens* group, are any differences that may exist between *w*PipMol and *w*PipPel where *w*PipPel and *w*PipJHB are identical. The *w*PipPel and *w*PipMol genomes proved to be highly similar and the great majority of genes have identical sequences; only thirty-three non-synonymous SNPs (single nucleotide polymorphisms) were identified in total, in twenty-three genes (Table S2, S3). However, three genes in three inserted or deleted regions were present in *w*PipMol but absent in *w*PipPel and *w*PipJHB (Table 3). One of these newly identified genes, *ankM1*, encodes ankyrin (ANK) repeats, and *ankM2* encodes ANK and Tetratricopeptide (TPR) repeats, both of which function in mediating protein-protein interactions [34]–[36]. The *ankM1* gene is a homolog of WD0596 from *Wolbachia* strain *w*Mel of *Drosophila melanogaster* [37]. The other newly identified gene, labeled *wtrM*, contains DNA binding domains associated with transcriptional regulators. Five inserted or deleted regions each comprising one to five genes were identified in *w*PipPel and *w*PipJHB but absent in *w*PipMol (table 3), all of which are within or adjacent to prophage regions.

**Table 3:** Inserted or deleted regions identified in the *w*PipMol genome that are absent from *w*PipPel (1–3), or present in the *w*PipPel genome but absent from *w*PipMol (4–7).

Indel (WO location)	Gene	Putative protein function/domain
1	*ank*M1	Ankyrin repeat motif; similar to WD0596
2	*ank*M2	Ankyrin and TPR repeat motif
3	*wtrM*	Transcriptional regulator
4 (WO3 end)	WP0348–WP0351	All hypothetical
5 (WO4)	WP0453	Hypothetical; transmembrane helix
	WP0454	Endonuclease
	WP0455	Patatin family phospholipase
	WP0456	Hypothetical; membrane lipoprotein attachment site
	WP0457	Transcriptional regulator
	WP0458	Hypothetical
6 (WO5 end)	WP1286–WP1288	All hypothetical
7 (WO5)	WP1337	Ankyrin repeat motif (ank57); Transmembrane domain

**Figure 5:**
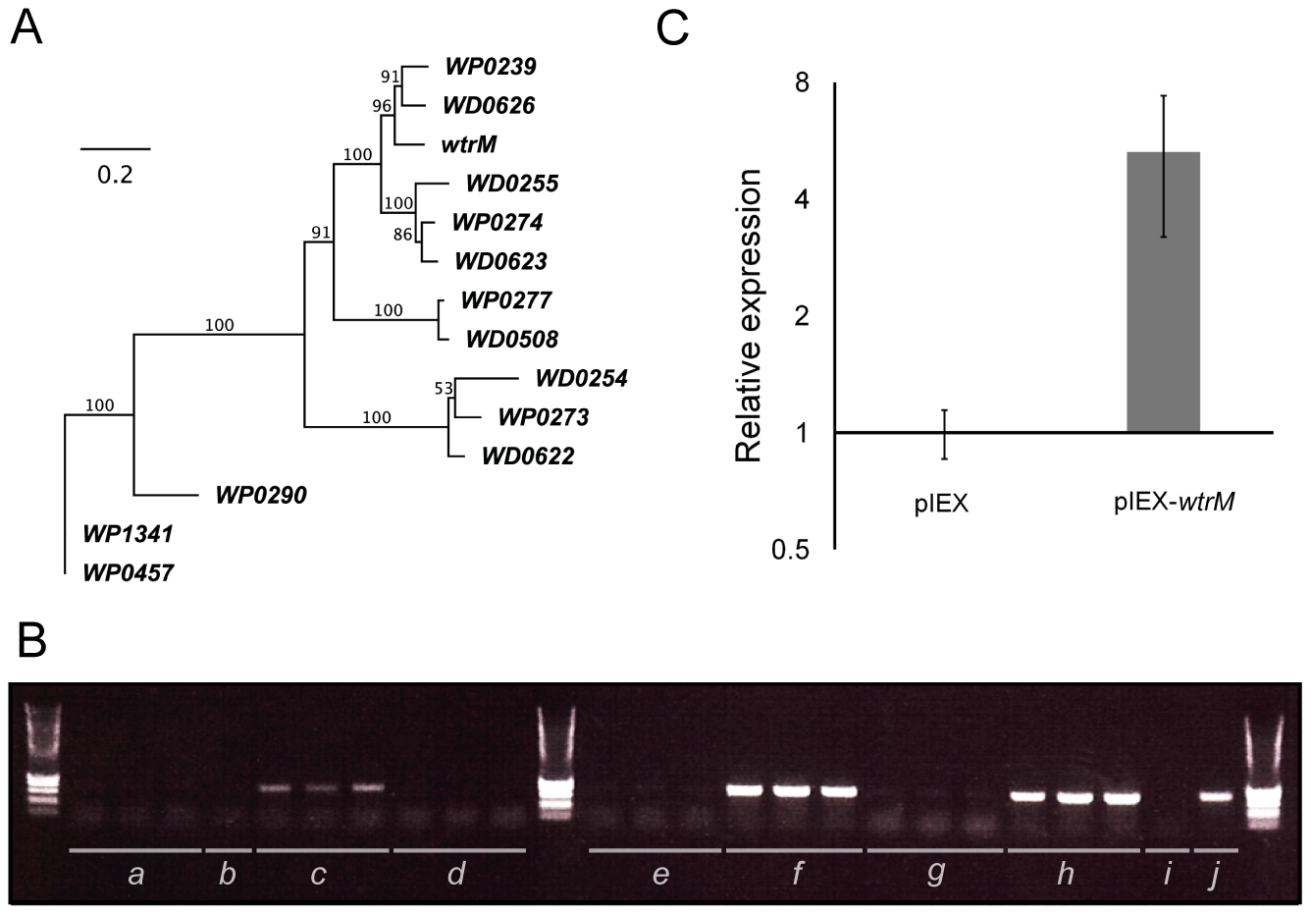
Analysis of transcription factor *wtrM.* **A**. Phylogenetic tree of *Wolbachia* transcriptional regulator (*wtr*) genes. An unrooted maximum likelihood phylogenetic tree, constructed using PHYLIP, of transcriptional regulator genes in the *w*Pip and *w*Mel genomes from amino acid alignments. Bootstrap percentages for 100 trees are shown for each node. **B**. Transcription of *wtrM* in *Culex* mosquitoes. Expression of *wtrM* was validated by RNA extraction and PCR on the cDNA using primers internal to the open reading frame of the gene. There is no transcription of *wtrM* detectable in control untransfected ovaries (a) or carcasses (b) from Pel. There is a high level of transcription of *wtrM* in ovaries from untransfected Mol mosquitoes (c) but not in carcasses (d). In Pel mosquitoes transfected with *wtrM* by injection into the thorax (e, f) there is no transcription detected in ovaries (e) but strong expression in carcasses (f). Transfection of *wtrM* into the abdomen of Pel mosquitoes produced no detectable transcription in ovaries (g) but transcription readily detected in carcasses (h). An untransfected whole female of Pel (i) and Mol (j) are also shown. **C**. *CPIJ005623* upregulation in transfected *Culex* females. Transcription of the *Culex* gene *CPIJ005623* in Pel females transfected with the *w*PipMol gene *wtrM*, compared to Pel females transfected with the same plasmid but containing no insert. Difference was significant at *p*<0.01 using a Wilcoxon rank sum test.

As a preliminary guide to which of the inserted or deleted regions most warranted further experimental investigation, we examined patterns of presence or absence in some other *C. pipiens* group populations from different geographical locations using PCR (Table S4). The *Wolbachia* transcriptional regulator gene *wtrM* was the only gene that occurred solely in the two *C. molestus* lines examined, Mol and Italy, which generate bidirectional CI with a selection of other *C. pipiens* group line with which they have been crossed (Table S1). This gene is a member of a family of *Wolbachia* transcriptional regulator genes, with seven genes in the *w*PipPel genome and six in the *D. melanogaster w*Mel *Wolbachia* genome (Figure 5A). One of the genes missing in *w*PipMol but present in *w*PipPel, WP0457, is also a member of this family of transcriptional regulators. WP0457 is identical to WP1341, located in a highly similar cluster of prophage-associated genes. Both of these loci are located close to patatin family phospholipase genes, which encode bacterial virulence factors that can disrupt cell membranes [38], [39].

### Transfection of *Wolbachia* gene *wtrM* into *Culex* females

We hypothesised that the protein products of the *Wolbachia* transcriptional regulator *wtrM* might be secreted, translocated to the host nucleus and modify host gene transcription as a means to manipulate the host. To investigate this possibility, in the absence of a transformation system for *Wolbachia*, host transfection was used. In the Mol line (*w*PipMol-infected) *C. molestus* mosquitoes, expression of *wtrM* was strongly localised to the ovaries in females (Figure 5B), which is where the majority of *Wolbachia* are located and are the important tissues with respect to CI. The *wtrM* gene was cloned into an insect expression plasmid and transfected into females of Pel *C. quinquefasciatus* in order to examine whether CI-like phenotypes could be induced; expression or translocation of WtrM protein in the ovaries would be required for this purpose. The transcription of *wtrM* was confirmed using RT-PCR in whole females transfected by intra-thoracic injection; however the transcript could not be detected in dissected ovaries, suggesting that the transfected plasmid did not cross the Peritoneal Sheath and enter the ovary (Figure 5B). Using quantitative RT-PCR, levels of transcription of *CPIJ005623* were then compared with control females transfected with plasmid that did not contain *wtrM*. There was more than five-fold up-regulation of *CPIJ005623* in *wtrM*-transfected females compared to the females injected with the plasmid alone (*p*<0.01, Wilcoxon rank sum test) (figure 5C).

## Discussion

Several lines of evidence support the hypothesis that the differential regulation of a Culex homolog of the D. melanogaster gene grau plays a direct role in CI generation. The presence of Wolbachia induced changes in CPIJ005623 transcription levels and the degree of upregulation of CPIJ005623 varied between incompatible C. pipiens group females. A decrease in embryo hatch rates was detected in a C. molestus line when CPIJ005623 expression was knocked down in females of an otherwise compatible cross. This increase in levels of CI suggests a role for CPIJ005623 in CI rescue in C. molestus females. The knockdown of CPIJ005623 also extended the mean developmental stage reached by embryos in incompatible crosses. When reversal of CI might have been expected (male KDs), more embryos arrested at a later stage and when increased levels of CI might have been expected (female KD), more embryos arrested at an early stage.

It has been hypothesized based on its mutant-sterile phenotype that D. melanogaster grau could be involved in egg activation, act as a negative regulator of the cyclin B complex stabilizing factor involved in meiotic cell cycle regulation, and/or regulate microtubules; these are not mutually exclusive possibilities [26]. Molecular changes that could affect cell cycle regulation fit with the known cell biology of CI [2]–[5]. Differential transcription leading to inappropriate levels of GRAU protein in the presence of Wolbachia during spermatogenesis/oogenesis could therefore be causally linked to the fact that cell cycle events in the male and female pronuclei are asynchronous.

Given that there was no complete reversal of embryo hatch rates in the rescue cross following CPIJ005623 knockdown, it is likely that differential regulation of CPIJ005623 is a component of CI but not the only molecular change that underlies the phenotype. It also seems likely that different Wolbachia strains produce different combinations of molecular manipulations, given the mutual incompatibilities that can exist among Wolbachia strains. The differences in grau homolog upregulation and KD analysis between the two C. pipiens group lines presented in this study reflect that. Further work is needed to determine what other factors along with grau are involved in this complex phenotype and whether other completely independent pathways are targeted in different Wolbachia strain-host associations where CI is induced. In light of the female-sterile phenotype produced by grau mutations, it would also be interesting to search for and examine the expression of grau homologs in Asobara tabida, where Wolbachia has become essential for the completion of this wasp's oogenesis [40]–[42].

The identification of a *Wolbachia* gene that modifies transcription levels of a non-immune host gene is a significant step forward in understanding the molecular methods by which this endosymbiont manipulates its hosts. This *Wolbachia* gene is one of only a small number that vary between two incompatible populations. Although it was not possible to directly assay the effects of *wtrM* on crossing patterns using transfection, since the plasmid did not enter the ovaries, the upregulation of CPIJ005623 in *wtrM*-transfected females does provide clear support for the hypothesis that *wtrM* has a role in the generation and/or rescue of CI between these *Culex* lines. The fact that the natural expression levels of *wtrM* in *C. molestus* were highest in ovaries is of interest given that CPIJ005623 knockdown in *C. molestus* females changed embryo development and hatch rates whereas CPIJ005623 knockdowns in males produced little effect. It should be noted that although *wtrM* appears to play a role in this particular *Wolbachia* - host interaction, there may well be a number of other *Wolbachia* genes beyond this family of homologous transcriptional regulators that interact directly with host genomes.

The small number of differences between the genomes of incompatibility-generating *w*Pip strains makes this a promising system for better elucidating the molecular mechanism of CI. All the genes identified here that vary between mutually incompatible *w*Pip substrains provide new avenues for investigation; their concentration in the *w*Pip prophages also underlines the importance of these hypervariable regions in *w*Pip recent genome evolution [13]. Ultimately, the development of a methodology for *Wolbachia* transformation may be required in order to produce a direct phenotypic confirmation of implicated *Wolbachia* genes in generating/rescuing CI. Despite considerable effort this remains a highly challenging goal, since it is an obligately intracellular microbe. Transformation of the host insect to express *Wolbachia* genes could be carried out, as was performed in *Drosophila* with several *Wolbachia* Ankyrin repeat-encoding genes [43]. However, given the possible differences in the activity of proteins when expressed in host cells rather than *Wolbachia*, for example in post-translational processing, folding and in epistatic interactions with other *Wolbachia* proteins within the bacteria, failure to generate a sterility phenotype does not rule out the involvement of candidate genes in CI.

Recently, Wolbachia has attracted considerable scientific and public interest as a control tool for mosquito-borne diseases. In the *Aedes* mosquito vectors of dengue virus, the introduction of several non-native *Wolbachia* strains produced a strong inhibitory effect on dengue virus transmission. Given its population invasion capacity, *Wolbachia*-based strategies seem very promising as a new dengue control tool [16], [44]–[47]. A better understanding of the molecular basis of CI has implications for the effectiveness of Wolbachia-based disease control strategies. It would assist in the design of multi-strain superinfections for spread into wild Wolbachia-infected populations or, in the event of the development of pathogen resistance, for achieving successive invasions of different Wolbachia strains [48]. Furthermore, knowledge of the host genes involved in this process could also aid the development of gene drive systems for spreading nuclear genes through pest insect populations [49].

## Materials and Methods

### Mosquito rearing, antibiotic treatment and mass crosses

C. pipiens complex laboratory lines Pel (Wolbachia infected C. quinquefasciatus, Sri Lanka) [13], Cpq (Wolbachia infected) and Cpq-T (Wolbachia uninfected C. quinquefasciatus USA) (kind gift from R. Glazer) [50], and Italy (Wolbachia infected) C. molestus line (kind gift from M. Petridis, collected in 1991 in Granarolo-Italy, by A. Medici and G. Rossi) were reared using standard mosquito rearing procedures at low larval densities in standard insectary conditions (27°C, 70% relative humidity) with a 12 h light/dark cycle.

Wolbachia uninfected PelU was generated by treating PelA with rifampicin (1 ml of 2.5 mg/ml added every 3 days in roughly 1 L H_2_0) throughout larval development for 5 consecutive generations. Adults were not treated with antibiotic. The line was subsequently reared in the absence of antibiotic for at least 5 generations more before experiments were performed. Removal of Wolbachia was confirmed by PCR and the line has been kept Wolbachia free since 2011. Cpq-T generation is described in detail in [50]. In brief Cpq adults were treated for one week on 1 mg/ml tetracycline (pH 7) in 10% sucrose. Wolbachia removal was confirmed by PCR and the line has been kept *Wolbachia* free since 2010.

For crosses conducted to establish patterns of compatibility between lines, mass crosses containing 50 individuals of each sex were set up, blood fed at three to five days post eclosion and embryos collected four days later; hatch of at least 800 embryos were scored per cross [13]. Where there was no embryo hatch from a cross, the experiment was repeated and females separated into individual containers for egg laying, and spermathecae then dissected to ensure that only progeny from inseminated females were scored.

### Genomic (g)DNA extractions

Adult mosquitoes were homogenized in STE buffer (10 mM Tris-HCl, 1 mM EDTA and 100 mM NaCl) and incubated for 10 min at 95–99°C. Samples were centrifuged for 5 minutes at maximum speed at 4°C. Supernatant, containing gDNA, was kept and used for Polymerase Chain Reaction (PCR); all oligonucleotides are listed in table S5.

### RNA isolation

Adult female and male RNA was extracted from 4–5 adult mosquitoes using TRIzol Reagent (Invitrogen-Life Technologies) following manufacturer's instructions. TRIzol-extracted RNA was DNase I treated for genomic DNA elimination and purified via standard phenol chloroform extraction. RNA from both mosquito ovaries and testes (30 individuals for each sample), was extracted and purified via the RNeasy Mini protocol for animal tissue (Qiagen). All purified RNA samples were quality checked via Nanodrop analysis and only highly pure (A260:230 and A260:280 ratios of >2.00) were kept. Exception was made for testes RNA for which the maximum A260:230 achieved were 1∶50-1.80 even when using a highly optimised protocol.

### cDNA synthesis and qRT-PCR

cDNA synthesis was performed in 10 µl (adults) or 20 µl (ovaries and testes) reaction volumes with 100, 300 or 900 ng of total RNA for the testes, ovaries and adult (male or female) samples respectively, using the iScript cDNA synthesis kit (BioRad). qRT-PCRs were performed on 1∶10, 1∶20 and 1∶30 dilutions of the cDNAs from the testes, ovaries and adult samples respectively, using iQ SYBR Green PCR mastermix (BioRad), a DNA Engine thermocycler (MJ Research) with a Chromo4 real-time PCR detection system (Bio-Rad) and the following cycling conditions: 95°C for 3 minutes, then 41 cycles of 95°C for 10 s, 60°C for 30 s, with fluorescence acquisition at the end of each cycle and a melting curve analysis after the final cycle. The cycle threshold (C_t_) values were determined and background fluorescence was subtracted. Transcription levels of target genes were calculated by the standard curve method, as described in technical bulletin #2 of the ABI Prism 7700 Manual (Applied Biosystems-Life Technologies), relative to the endogenous reference genes and RpS7.

### Gene knockdown experiments

T7-tailed primers were used to amplify a PCR template for CPIJ005623 and the control LacZ gene from Culex cDNA and E.coli DNA respectively. DsRNAs were synthesized using the T7 Megascript Kit (Ambion-Life Technologies) following manufacturer's advice. Purified dsRNAs were diluted to a concentration of 3 µg/µl and 69 nl were injected into the thorax of cold anesthetized mosquitoes using a Nanoject microinjector (Drummond Scientific). After dsRNA injections and at the indicated time points, testes and ovaries from 30 male and female mosquitoes were dissected. RNA was extracted by using the RNeasy Mini protocol for animal tissue (Qiagen) in order to verify the successful knockdown of the *CPIJ005623* transcript. Amplifications were performed using primers *RpL32* (F-R) and *CPIJ005623* (F1-R1) for *Culex* Pel, *RpLS7* and *CPIJ005623* (F3-R3) for *Culex* Italy as described above.

Crossing experiments following knockdowns were carried out using 50 virgin individuals of each sex obtained by sexing and isolating pupae for mosquitoes. On the day of pupal eclosion, 50 male or 50 female mosquitoes were injected with dsRNA. Injected individuals were allowed to recover and on the dates represented in the experimental design diagrams mating partners were added to the injected individuals for the designated times indicated (Fig. 3A, 4A and 5A). After egg lay, female spermathecae were examined for the presence of sperm if the hatch rate was zero to confirm insemination and only progeny of inseminated females were scored. The F_1_ generation progeny from crosses were characterised by i) total number of rafts; ii) total number of eggs, iii) mean proportion of hatched eggs and iv) for unhatched embryos mean proportion of developed embryos.

### Statistical analysis

Transcription levels were analysed using a non-parametric Wilcoxon rank-sum test in the case of 2 sample variables and by a two-way ANOVA (with replication) analysis of variance for 3 sample variables. Embryo developmental results were analysed by using generalized linear models (GLM) with binomial error structures (where reaching SII or beyond is classed as a success). Differences between hatched and unhatched embryos were determined via a Welch two sample t-test. All calculations were performed using the R software (R Development Core Team, 2004).

### Genome assembly and SNP identification

The purification of *Wolbachia w*Pip DNA from Mol early embryos, library construction, general PCR conditions and crossing experiments were as previously described [10]. The genome was assembled from three independent 454 runs. One half-plate from a standard single-end FLX library generated 14 Mb of data from 80,752 reads, with an average length of 174 bp. Two quarter-plates from a similar library generated 30.9 Mb of data from 80,954 reads with an average length of 264 bp. Finally one quarter-plate from a 3.2 kb paired-end Titanium library generated 48.6 Mb from 157,851 reads with an average length of 232 bp. Together these runs produced a total of 93.5 Mb of data, which was combined and assembled with Newbler (Roche), using default parameters. The final assembly gave 194 contigs >500 bp with a mean coverage of 41× and N50 of 19.5 kb, linked into 24 scaffolds plus 79 unscaffolded contigs, with an N50 of 567 kb, and a total length of 1,435,676 bp. 99.6% of bases had a quality value >Q40. The sequences generated have been submitted to the EMBL/GenBank/DDBJ database with the accession number HG428761.

Phylogenetic trees were constructed based on Maximum Likelihood using PhyML [51], using the Whelan and Goldman substitution model, on an amino acid alignment created with ClustalW; one hundred data sets were generated for bootstrapping.

Contigs and scaffolds were mapped against the *w*Pip genome as a reference using mummer. SNPs were called where the mapped sequences differed from the reference sequence. The contig and scaffold SNPs were mostly at contig boundaries and phage regions, where mapping was difficult. Only SNPs which were agreed upon by both the contig and scaffold mapping were considered strongly supported.

### PCR for SNP genotyping

For PCR analysis, genomic DNA was extracted from whole mosquitoes as above. Primers were designed to flank SNP regions and amplified using standard PCR conditions (94°C for 30 sec, 52 to 60°C for 30 sec and 72°C for 30 sec to 60 sec ×38 cycles). PCR products were purified using a Qiagen PCR purification kit and sequenced using GATC Biotech sequencing. The *C. pipiens* group lines used for these PCR experiments were as previously described [13], [28], [29] with an additional colony assayed from the Italy (Granarolo) collected in 1991.

### Construction of plasmids

The transcriptional regulator *wtrM* was PCR amplified from the strain of *Wolbachia* found in *Culex molestus*. DNA was extracted as above. PCR was conducted with Phusion *Taq* polymerase (Finnzymes) according to manufacturer's instructions. PCR products were digested and ligated into the NotI-SacI site of insect cell expression vector, pIEX8 (Novagen). Plasmids were purified using endotoxin free maxi preps (Qiagen).

### Transfection experiments

Adult *C. quinquefasciatus* Pel line mosquitoes less than 24 hours post eclosion, infected with *Wolbachia w*PipPel were intrathoracically injected with 200 ng plasmid DNA and Cellfectin II (Invitrogen) transfection reagent according to a previously published protocol [52]. Using a hand-held Nanoject (Drummond), adults were injected with either pIEX8-*wtrM* or a control plasmid with no insert and left to recover. Adults were isolated for total RNA extraction in groups of 2 to 4 individuals: 10 replicates of 3–4 individuals (n = 32) in pIEX and 10 replicates of 2–3 individuals (n = 27) for pIEX *wtrM*. cDNA synthesis was performed in 10 µl reaction volumes with 1000 ng of total RNA using the iScript cDNA synthesis kit (BioRad). qRT-PCRs were performed on 1∶20 dilutions of the cDNA using DyNAmo Colorflash (Fisher), a DNA Engine thermocycler (MJ Research) with a Chromo4 real-time PCR detection system (Bio-Rad) and the cycling conditions as above. Transcription levels were calculated by the standard curve method relative to the endogenous *Culex* reference gene *RpL32*.

## Supporting Information

Figure S1 Comparative analysis between CPIJ005623 and CPIJ015950. A. Schematic representation of the gene architecture of CPIJ005623 and CPIJ015950. The two predicted genes are virtually the same with the exception of 1 nucleotide (nt) change leading to a P-S substitution at the N-terminus of the predicted proteins, 1 nt deletion near the exon1-exon2 border of CPIJ015950 leading to two amino acid substitutions and the 56 nt deletion leading to the loss of a Zinc-finger C2H2 type domain signature (PS00028). B. Analysis of 56 nt indel region of CPIJ015950. Specific primers flanking the 56 nt deletion region of CPIJ015950 that amplify an 84 basepair (bp) and a 141 bp fragment of CPIJ015950 in predicted cDNA and gDNA, respectively and a 141 bp fragment of CPIJ005623 in predicted gDNA or cDNA were used in electrophoresis analysis following PCR. Only a fragment around 140 bp, consistent with the presence of only CPIJ005623, was amplified in these two C. pipiens group lines. RpS7 PCR with specific primers spanning an intron were run in parallel to check the quality of gDNA, cDNA and PCR reaction; No T = no template control.
(TIF)

Table S1 Crossing relationships between a selection of *C. pipiens* group lines (at least 800 embryos counted per cross); other crossing data between these lines are listed in table 1 and in previous publications [13], [28], [29].
(DOC)

Table S2 Genes containing at least one non- synonymous SNP when the *Wolbachia w*Pip Mol and Pel genomes are compared. The nucleotide sequence of each SNP is shown for Pel and Mol (column 5) and the amino acid that corresponds to that sequence is also shown (column 6). The nucleotide sequence of the SNPs in JHB is shown where the sequence is the same as either Pel or Mol.
(DOC)

Table S3 SNPs in Italy and Thai lines compared to Pel and Mol. Sequence data obtained for SNPs that showed the same nucleotide in JHB as for Pel were assessed in the Italy and Thai lines which have the same crossing types where tested as Mol and Pel/JHB respectively (table S1). The nucleotide at each SNP position is shown and whether it corresponds to the SNP sequence for Pel or Mol.
(DOC)

Table S4 PCR tests for presence (+) or absence (−) of inserted or deleted region genes that vary between the *w*PipPel and *w*PipMol genomes in a sample of *C. pipiens* group lines.
(DOC)

Table S5 List of oligonucleotides used.
(DOC)

## References

[1] Zug R, Hammerstein P (2012) Still a host of hosts for Wolbachia: analysis of recent data suggests that 40% of terrestrial arthropod species are infected. PLoS One 7: e38544. 22685581 10.1371/journal.pone.0038544 PMC3369835

[2] Serbus LR, Casper-Lindley C, Landmann F, Sullivan W (2008) The genetics and cell biology of Wolbachia-host interactions. Annu Rev Genet 42: 683–707. 18713031 10.1146/annurev.genet.41.110306.130354

[3] Callaini G, Riparbelli MG, Giordano R, Dallai R (1996) Mitotic defects associated with cytoplasmic incompatibility in Drosophila simulans. J Inv Path 67: 55–64.

[4] Callaini G, Dallai R, Riparbelli MG (1997) Wolbachia-induced delay of paternal chromatin condensation does not prevent maternal chromosomes from entering anaphase in incompatible crosses of Drosophila simulans. J Cell Sci 110: 271–80. 9044057 10.1242/jcs.110.2.271

[5] Landmann F, Orsi GA, Loppin B, Sullivan W (2009) Wolbachia-mediated cytoplasmic incompatibility is associated with impaired histone deposition in the male pronucleus. PLoS Pathog 5: e1000343. 19300496 10.1371/journal.ppat.1000343 PMC2652114

[6] Tram U, Sullivan W (2002) Role of delayed nuclear envelope breakdown and mitosis in Wolbachia-induced cytoplasmic incompatibility. Science 296: 1124–1126. 12004132 10.1126/science.1070536

[7] Riparbelli MG, Giordano R, Callaini G (2007) Effects of Wolbachia on sperm maturation and architecture in Drosophila simulans Riverside. Mech Dev 124: 699–714. 17693061 10.1016/j.mod.2007.07.001

[8] Laven H (1967) Speciation and evolution in Culex pipiens. In: Wright JW, Pal R, editors. Genetics of Insect Vectors of Disease. Amsterdam: Elsevier. pp. 251–275.

[9] Barr AR (1980) Cytoplasmic incompatibility in natural populations of a mosquito, *Culex pipiens* L. Nature 283: 71–72. 7350526 10.1038/283071a0

[10] Irving-Bell RJ (1983) Cytoplasmic incompatibility within and between *Culex molestus* and *Cx. quinquefasciatus* (Diptera: Culicidae). J Med Entomol 20: 44–48.

[11] Magnin M, Pasteur N, Raymond M (1987) Multiple incompatibilities within populations of Culex pipiens L. in southern France. Genetica 74: 125–130. 3506532 10.1007/BF00055223

[12] Guillemaud T, Pasteur N, Rousset F (1997) Contrasting levels of variability between cytoplasmic genomes and incompatibility types in the mosquito Culex pipiens. Proc Biol Sci 264: 245–251. 9061971 10.1098/rspb.1997.0035 PMC1688252

[13] Sinkins SP, Walker T, Lynd AR, Steven AR, Makepeace BL, et al (2005) Wolbachia variability and host effects on crossing type in Culex mosquitoes. Nature 436: 257–260. 16015330 10.1038/nature03629

[14] Duron O, Weill M (2006) Wolbachia infection influences the development of Culex pipiens embryos in incompatible crosses. Heredity 96: 493–500. 16639421 10.1038/sj.hdy.6800831

[15] Kambris Z, Cook PE, Phuc HK, Sinkins SP (2009) Immune activation by life- shortening Wolbachia and reduced filarial competence in mosquitoes. Science 326: 134–136. 19797660 10.1126/science.1177531 PMC2867033

[16] Moreira LA, Iturbe-Ormaetxe I, Jeffery JA, Lu G, Pyke AT, et al (2009) A Wolbachia symbiont in Aedes aegypti limits infection with dengue, chikungunya, and Plasmodium. Cell 139: 1268–1278. 20064373 10.1016/j.cell.2009.11.042

[17] Bian G, Xu Y, Lu P, Xie Y, Xi Z (2010) The endosymbiotic bacterium Wolbachia induces resistance to dengue virus in Aedes aegypti. PLoS Pathog 6: e1000833. 20368968 10.1371/journal.ppat.1000833 PMC2848556

[18] Kambris Z, Blagborough AM, Pinto SB, Blagrove M, Godfray HCJ, et al (2010) Wolbachia stimulates immune gene expression and inhibits Plasmodium development in Anopheles gambiae. PLoS Pathog 6: e1001143. 20949079 10.1371/journal.ppat.1001143 PMC2951381

[19] Hughes GL, Ren X, Ramirez JL, Sakamoto JM, Bailey JA, et al (2011) Wolbachia infections in Anopheles gambiae cells: transcriptomic characterization of a novel host-symbiont interaction. PLoS Pathog 7: e1001296. 21379333 10.1371/journal.ppat.1001296 PMC3040664

[20] Pan X, Zhou G, Wu J, Bian G, Lu P, et al (2012) Wolbachia induces reactive oxygen species (ROS)-dependent activation of the Toll pathway to control dengue virus in the mosquito Aedes aegypti. Proc Natl Acad Sci USA 109: E23–31. 22123956 10.1073/pnas.1116932108 PMC3252928

[21] Pinto SB, Mariconti M, Bazzocchi C, Bandi C, Sinkins SP (2012) Wolbachia Surface Protein induces innate immune responses in insect cells. BMC Microbiol 12: S11. 22375833 10.1186/1471-2180-12-S1-S11 PMC3287508

[22] Bian G, Joshi D, Dong Y, Lu P, Zhou G, Pan X, Xu Y, Dimopoulos G, Xi Z (2013) *Wolbachia* invades *Anopheles stephensi* populations and induces refractoriness to *Plasmodium* infection. Science 340: 748–751. 23661760 10.1126/science.1236192

[23] Clark ME, Heath BD, Anderson CL, Karr TL (2006) Induced paternal effects mimic cytoplasmic incompatibility in Drosophila. Genetics 173: 727–734. 16489228 10.1534/genetics.105.052431 PMC1526537

[24] Xi Z, Gavotte L, Xie Y, Dobson SL (2008) Genome-wide analysis of the interaction between the endosymbiotic bacterium Wolbachia and its Drosophila host. BMC Genomics 9: 1. 18171476 10.1186/1471-2164-9-1 PMC2253531

[25] Zheng Y, Ren PP, Wang JL, Wang YF (2011) Wolbachia-induced cytoplasmic incompatibility is associated with decreased Hira expression in male Drosophila. PLoS One 6: e19512. 21559343 10.1371/journal.pone.0019512 PMC3084885

[26] Page AW, Orr-Weaver TL (1996) The Drosophila genes grauzone and cortex are necessary for proper female meiosis. J Cell Science 109: 1707–1715. 8832393 10.1242/jcs.109.7.1707

[27] Chen B, Harms E, Chu T, Henrion G, Strickland S (2000) Completion of meiosis in Drosophila oocytes requires transcriptional control by grauzone, a new zinc finger protein. Development 127: 1243–1251. 10683177 10.1242/dev.127.6.1243

[28] Klasson L, Walker T, Sebaihia M, Sanders MJ, Quail MA, et al (2008) Genome evolution of *Wolbachia* strain *w*Pip from the *Culex pipiens* group. Mol Biol Evol 25: 1877–1887. 18550617 10.1093/molbev/msn133 PMC2515876

[29] Walker T, Klasson L, Sebaihia M, Sanders M, Thomson N, et al (2007) Ankyrin repeat domain-encoding genes in the *w*Pip strain of *Wolbachia* from the *Culex pipiens* group. BMC Biol 5: 39–47. 17883830 10.1186/1741-7007-5-39 PMC2045654

[30] Salzberg SL, Puiu D, Sommer DD, V. Nene V, Lee NH (2009) Genome sequence of the *Wolbachia* endosymbiont of *Culex quinquefasciatus* JHB. J Bacteriol 191: 1725. 19114486 10.1128/JB.01731-08 PMC2648186

[31] Arensburger P, Megy K, Waterhouse RM, Abrudan J, Amedeo P, et al (2010) Sequencing of Culex quinquefasciatus establishes a platform for mosquito comparative genomics. Science 330: 86–88. 20929810 10.1126/science.1191864 PMC3740384

[32] Wright JD, Barr AR (1981) Wolbachia and the normal and incompatible eggs of Aedes polynesiensis (diptera, Culicidae). J Invert Pathol 38: 409–418.

[33] Sharma VP, Hollingworth RM, Paschke JD (1970) Incorporation of tritiated thymidine in male and female mosquitoes, Culex pipiens with particular reference to spermatogenesis. J Insect Physiol 16: 429–436. 5438064 10.1016/0022-1910(70)90183-6

[34] Sedgwick SG, Smerdon SJ (1999) The ankyrin repeat: a diversity of interactions on a common structural framework. Trends Biochem Sci 24: 311–316. 10431175 10.1016/s0968-0004(99)01426-7

[35] Caturegli P, Asanovich KM, Walls JJ, Bakken JS, Madigan JE, et al (2000) ankA: an *Ehrlichia phagocytophila* group gene encoding a cytoplasmic protein antigen with ankyrin repeats. Infect Immun 68: 5277–5283. 10948155 10.1128/iai.68.9.5277-5283.2000 PMC101789

[36] D'Andrea LD, Regan L (2003) TPR proteins: the versatile helix. Trends Biochem Sci 28: 655–662. 14659697 10.1016/j.tibs.2003.10.007

[37] Wu M, Sun LV, Vamathevan J, Riegler M, Deboy R, et al (2004) Phylogenomics of the reproductive parasite *Wolbachia pipientis w*Mel: a streamlined genome overrun by mobile genetic elements. PLoS Biol 2: E69. 15024419 10.1371/journal.pbio.0020069 PMC368164

[38] Sato H, Frank DW (2004) ExoU is a potent intracellular phospholipase. Mol Microbiol 53: 1279–1290. 15387809 10.1111/j.1365-2958.2004.04194.x

[39] Rahman MS, Ammerman NC, Sears KT, Ceraul SM, Azad AF (2010) Functional characterization of a phospholipase A(2) homolog from *Rickettsia typhi* . J Bacteriol 192: 3294–3303 (2010). 20435729 10.1128/JB.00155-10 PMC2897650

[40] Dedeine F, Vavre F, Fleury F, Loppin B, Hochberg ME, et al (2001) Removing symbiotic Wolbachia bacteria specifically inhibits oogenesis in a parasitic wasp. Proc Natl Acad Sci USA 98: 6247–6252. 11353833 10.1073/pnas.101304298 PMC33453

[41] Dedeine F, Boulétreau M, Vavre F (2005) Wolbachia requirement for oogenesis: occurrence within the genus *Asobara* (Hymenoptera, Braconidae) and evidence for intraspecific variation in *A. tabida* . Heredity 95: 394–400. 16118660 10.1038/sj.hdy.6800739

[42] Kremer N, Dedeine F, Charif D, Finet C, Allemand R, et al (2010) Do variable compensatory mechanisms explain the polymorphism of the dependence phenotype in the Asobara tabida-Wolbachia association? Evolution 64: 2969–2979. 20482609 10.1111/j.1558-5646.2010.01034.x

[43] Yamada R, Iturbe-Ormaetxe I, Brownlie JC, O'Neill SL (2011) Functional test of the influence of *Wolbachia* genes on cytoplasmic incompatibility expression in *Drosophila melanogaster* . Insect Mol Biol 20: 75–85. 20854481 10.1111/j.1365-2583.2010.01042.x

[44] McMeniman CJ, Lane RV, Cass BN, Fong AW, Sidhu M, et al (2009) Stable introduction of a life-shortening Wolbachia infection into the mosquito Aedes aegypti. Science 323: 141–144. 19119237 10.1126/science.1165326

[45] Walker T, Johnson PH, Moreira LA, Iturbe-Ormaetxe I, Frentiu FD, et al (2011) The wMel Wolbachia strain blocks dengue and invades caged Aedes aegypti populations. Nature 476: 450–453. 21866159 10.1038/nature10355

[46] Hoffmann AA, Montgomery BL, Popovici J, Iturbe-Ormaetxe I, Johnson PH, et al (2011) Successful establishment of Wolbachia in Aedes populations to suppress dengue transmission. Nature 476: 454–457. 21866160 10.1038/nature10356

[47] Blagrove MS, Arias-Goeta C, Failloux A-B, Sinkins SP (2012) The Wolbachia strain wMel induces cytoplasmic incompatibility and blocks dengue transmission in Aedes albopictus. Proc Natl Acad Sci USA 109: 255–60. 22123944 10.1073/pnas.1112021108 PMC3252941

[48] Sinkins SP, Gould F (2006) Gene drive systems for insect disease vectors. Nat Rev Gen 7: 427–435. 10.1038/nrg1870 16682981

[49] Sinkins SP, Godfray HCJ (2004) Use of Wolbachia to drive nuclear transgenes through insect populations. Proc Biol Sci 271: 1421–1426. 15306342 10.1098/rspb.2004.2740 PMC1691734

[50] Glaser RL, Meola MA (2010) The native Wolbachia endosymbionts of Drosophila melanogaster and Culex quinquefasciatus increase host resistance to West Nile virus infection. PLoS One 5: e11977. 20700535 10.1371/journal.pone.0011977 PMC2916829

[51] Guindon S, Gascuel O (2003) A simple, fast, and accurate algorithm to estimate large phylogenies by maximum likelihood. Syst Biol 52: 696–704. 14530136 10.1080/10635150390235520

[52] Cheng G, Liu L, Wang P, Zhang Y, Zhao YO, et al (2011) An *in vivo* transfection approach elucidates a role for *Aedes aegypti* thioester-containing proteins in flaviviral infection. PLoS One 6: e22786. 21818390 10.1371/journal.pone.0022786 PMC3144946

