# Supplementary figures and images for: Transcriptional Regulation of Culex pipiens Mosquitoes by Wolbachia Influences Cytoplasmic Incompatibility
Source: PLoS Pathog. 2013 Oct 31;9(10):e1003647. doi: 10.1371/journal.ppat.1003647 (PMC3814344; doi:10.1371/journal.ppat.1003647)

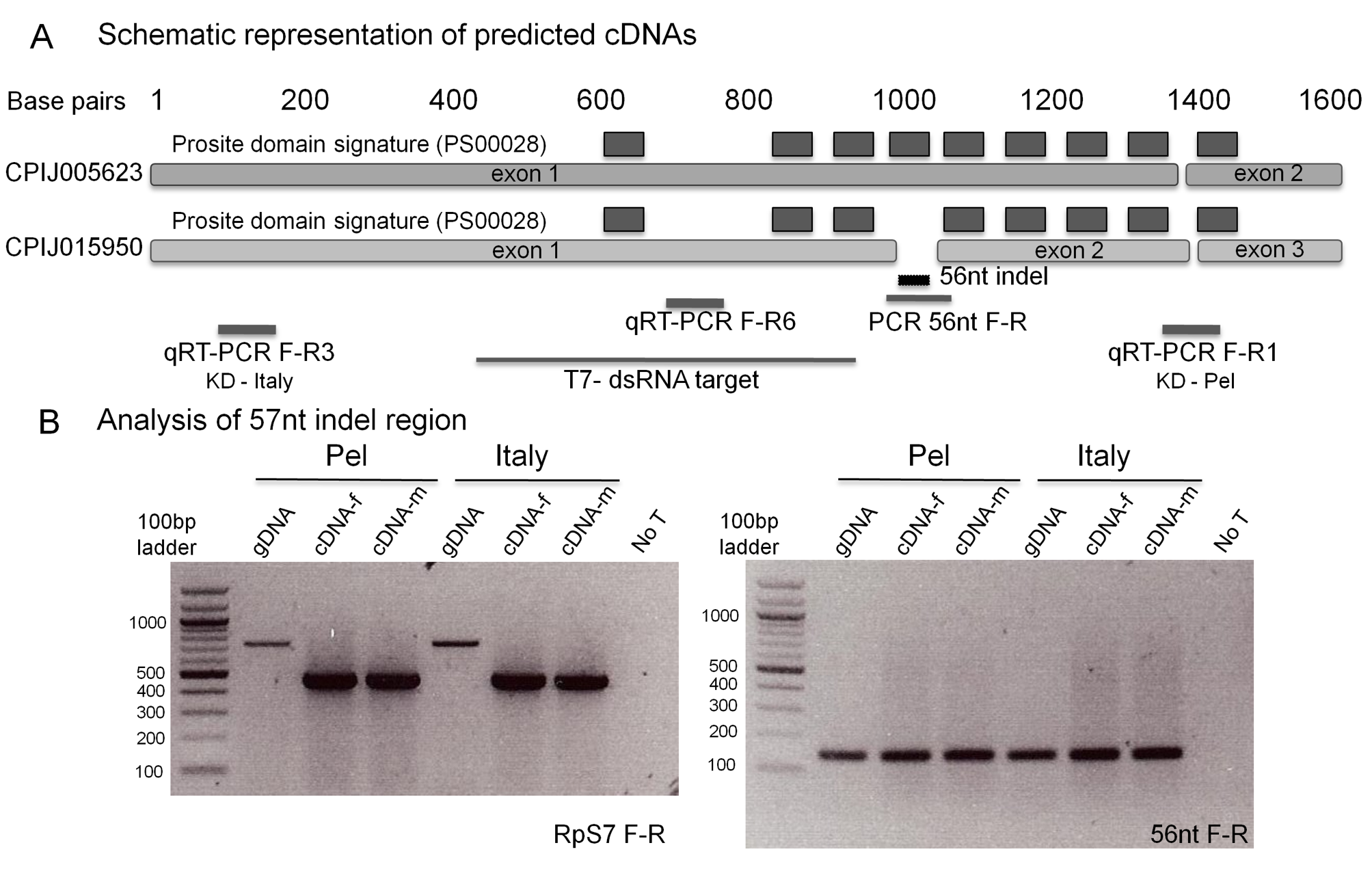

Supplement: Figure S1 — Comparative analysis between CPIJ005623 and CPIJ015950. A. Schematic representation of the gene architecture of CPIJ005623 and CPIJ015950. The two predicted genes are virtually the same with the exception of 1 nucleotide (nt) change leading to a P-S substitution at the N-terminus of the predicted proteins, 1 nt deletion near the exon1-exon2 border of CPIJ015950 leading to two amino acid substitutions and the 56 nt deletion leading to the loss of a Zinc-finger C2H2 type domain signature (PS00028). B. Analysis of 56 nt indel region of CPIJ015950. Specific primers flanking the 56 nt deletion region of CPIJ015950 that amplify an 84 basepair (bp) and a 141 bp fragment of CPIJ015950 in predicted cDNA and gDNA, respectively and a 141 bp fragment of CPIJ005623 in predicted gDNA or cDNA were used in electrophoresis analysis following PCR. Only a fragment around 140 bp, consistent with the presence of only CPIJ005623, was amplified in these two C. pipiens group lines. RpS7 PCR with specific primers spanning an intron were run in parallel to check the quality of gDNA, cDNA and PCR reaction; No T = no template control. (TIF) [file ppat.1003647.s001.tif]
