# Supplementary material for: Transcriptional Regulation of Culex pipiens Mosquitoes by Wolbachia Influences Cytoplasmic Incompatibility
Source: PLoS Pathog. 2013 Oct 31;9(10):e1003647. doi: 10.1371/journal.ppat.1003647 (PMC3814344; doi:10.1371/journal.ppat.1003647)
Supplement: Table S1 — Crossing relationships between a selection of C. pipiens group lines (at least 800 embryos counted per cross); other crossing data between these lines are listed in table 1 and in previous publications [13], [28], [29]. (DOC) [file ppat.1003647.s002.doc]

**Table S1**

| Cross | % hatch | Cross | % hatch | compatibility |
| --- | --- | --- | --- | --- |
| Pel♀ x JHB♂ | 84 | JHB♀ x Pel♂ | 87 | compatible |
| Mol♀ x Pel♂ | 0 | Pel ♀x Mol♂ | 0 | bidirectional CI |
| Mol♀ x JHB♂ | 0 | JHB♀ x Mol♂ | 0 | bidirectional CI |
| Pel♀ x Muh♂ | 73 | Muh♀ x Pel♂ | 79 | compatible |
| Pel♀ x Italy♂ | 0 | Italy♀ x Pel♂ | 0 | bi-directional CI |
| Mol ♀x Italy♂ | 91 | Italy♀ x Mol♂ | 85 | compatible |
