# Supplementary material for: Transcriptional Regulation of Culex pipiens Mosquitoes by Wolbachia Influences Cytoplasmic Incompatibility
Source: PLoS Pathog. 2013 Oct 31;9(10):e1003647. doi: 10.1371/journal.ppat.1003647 (PMC3814344; doi:10.1371/journal.ppat.1003647)
Supplement: Table S2 — Genes containing at least one non- synonymous SNP when the Wolbachia wPip Mol and Pel genomes are compared. The nucleotide sequence of each SNP is shown for Pel and Mol (column 5) and the amino acid that corresponds to that sequence is also shown (column 6). The nucleotide sequence of the SNPs in JHB is shown where the sequence is the same as either Pel or Mol. (DOC) [file ppat.1003647.s003.doc]

**Table S2.**

| **Locus tag** | **Closest *w*Mel**  **locus tag** | **Product function / domain**  **(phage if applicable)** | **SNP position in**  **Gene** | **Nucleotide (pel>mol)** | **Amino acid (pel>mol)** | **SNP in**  **JHB** |
| --- | --- | --- | --- | --- | --- | --- |
| WP0035 | WD0332 | hypothetical protein | 1390 | G>A | (A>T) | A (Mol) |
| WP0082 | WD0301 | CoxA - cytochrome c oxidase I | 37 | C>T | (L>F) | T (Mol) |
| WP0140 | WD0928 | chaperone protein dnak - hsp70 | 876 | C>G | (I>M) | G (Mol) |
| WP0278 | WD0509 | DNA repair protein MutL (WO-1) | 958 | A>C | (I>L) | absent |
| WP0292 | WD0512 | ankyrin repeat domain protein  (adjacent WO-2) | 3665 | C>T | (A>V) | A (Pel) |
| WP0297 | WD0634 | site-specific recombinase,  resolvase family (WO-pip2) | 1. 1373  2. 1376  3. 1379  4. 1385  5. 1388  6. 1391  7. 1393 | A>T  A>G  T>G  A>G  G>A  G>T  C>G | (E>V)  (D>G)  (I>R)  (N>S)  (G>E)  (R>M)  (Q>E) | A (Pel)  A (Pel)  T (Pel)  A (Pel)  G (Pel)  G (Pel)  C (Pel) |
| WP0423 | none | glyoxalase/bleomycin resistance protein/dioxygenase (WO-4) | 302 | G>T | (R>M) | A |
| WP0430 | WD0262 | putative phage related protein  (WO-4) | 1. 175  2. 355  3. 358-60 | C>T  G>A  AAG>CGT | (L>F)  (V>I)  (K>R) | T (Mol)  G (Pel)  AAG (Pel) |
| WP0491 | WD0500 | type II secretion system protein,  putative | 832 | A>G | (K>E) | G (Mol) |
| WP0502 | WD0762 | peptidase, M16 family | 275 | G>A | (R>K) | A (Mol) |
| WP0512 | none | hypothetical protein | 1240 | C>G | (Q>E) | C (Pel) |
| WP0599 | WD0854 | TrlB/VirB6 plasmid conjugal transfer | 1. 271  2. 2606 | G>A  G>A | (G>S)  (G>E) | G (Pel)  G (Pel) |
| WP0666 | none | hypothetical protein; probable  transmembrane helix | 761 | C>T | (A>V) | C (Pel) |
| WP0669 | WD0868 | pgm phosphoglycerate mutase, 2,3-bisphosphoglycerate-independent | 1344 | G>C | (Q>H) | C (Mol) |
| WP0713 | none | hypothetical protein | 166 | C>T | (L>F) | C (Pel) |
| WP0717 | WD1136 | iron compound ABC transporter, permease | 394 | A>G | (K>E) | A (Pel) |
| WP0752 | WD1187 | putative membrane protein | 473 | G>T | (C>F) | C (Pel) |
| WP0753 | WD1188 | glutamate-cysteine ligase-related | 79 | C>T | (P>S) | C (Pel) |
| WP0848 | WD1128 | murF - cell envelope biogenesis,  outer membrane | 1348 | G>A | (V>I) | G (Pel) |
| WP0903 | WD1249 | glutathione-regulated potassium-efflux | 1264 | A>G | (I>V) | G (Mol) |
| WP0935 | WD1065 | hypothetical protein | 319 | A>G | (R>G) | A (Pel) |
| WP1050 | WD0818 | hypothetical protein | 1 | G>A | (V>M) | G (Pel) |
| WP1336 | WD0567 | GpD phage late control d protein  (WO-5) | 416 | C>T | (A>V) | C (Pel) |
