# Supplementary material for: Transcriptional Regulation of Culex pipiens Mosquitoes by Wolbachia Influences Cytoplasmic Incompatibility
Source: PLoS Pathog. 2013 Oct 31;9(10):e1003647. doi: 10.1371/journal.ppat.1003647 (PMC3814344; doi:10.1371/journal.ppat.1003647)
Supplement: Table S3 — SNPs in Italy and Thai lines compared to Pel and Mol. Sequence data obtained for SNPs that showed the same nucleotide in JHB as for Pel were assessed in the Italy and Thai lines which have the same crossing types where tested as Mol and Pel/JHB respectively (table S1). The nucleotide at each SNP position is shown and whether it corresponds to the SNP sequence for Pel or Mol. (DOC) [file ppat.1003647.s004.doc]

**Table S3.**

| **locus tag** | **Italy** | **Thai** |
| --- | --- | --- |
| WP0292 | T (Mol) | T (Mol) |
| WP0297 | 1. A (Pel)  2. A (Pel)  3. T (Pel)  4. A (Pel)  5. G (Pel)  6. G (Pel)  7. C (Pel) | A (Pel)  A (Pel)  T (Pel)  A (Pel)  G (Pel)  G (Pel)  C (Pel) |
| WP0430 | 1. T (Mol)  2. A (Mol)  3. C (Mol)  4. T (Mol)  5. T (Mol) | T (Mol)  A (Mol)  C (Mol)  T (Mol)  T (Mol) |
| WP0512 | G (Mol) | C(Pel) |
| WP0599 | 1. A (Mol)  2. A (Mol) | G (Pel)  G (Pel) |
| WP0666 | T (Mol) | C (Pel) |
| WP0713 | T (Mol) | T (Mol) |
| WP0717 | G (Mol) | G (Mol) |
| WP0752 | T (Pel) | G (Mol) |
| WP0753 | T (Mol) | C (Pel) |
| WP0848 | A (Mol) | A (Mol) |
| WP0935 | G (Mol) | G (Mol) |
| WP1050 | A (Mol) | A (Mol) |
| WP1336 | T (Mol) | T (Mol) |
