# Supplementary material for: Transcriptional Regulation of Culex pipiens Mosquitoes by Wolbachia Influences Cytoplasmic Incompatibility
Source: PLoS Pathog. 2013 Oct 31;9(10):e1003647. doi: 10.1371/journal.ppat.1003647 (PMC3814344; doi:10.1371/journal.ppat.1003647)
Supplement: Table S4 — PCR tests for presence (+) or absence (−) of inserted or deleted region genes that vary between the wPipPel and wPipMol genomes in a sample of C. pipiens group lines. (DOC) [file ppat.1003647.s005.doc]

**Table S4.**

|  | 1  *ank*M1 | 2  *ank*M3 | 3  *wtr*M | 4 wp0348 | 5 wp0455 | 6 wp1286 | 7 wp1337 |
| --- | --- | --- | --- | --- | --- | --- | --- |
| Mol | + | + | + | - | - | - | - |
| Pel | - | - | - | + | + | + | + |
| JHB | - | - | - | + | + | + | + |
| Col | + | + | - | + | - | + | - |
| Muh | - | - | - | + | + | + | + |
| Thai | - | - | - | + | - | + | - |
| Italy | + | + | + | - | - | - | - |
