# Supplementary material for: Transcriptional Regulation of Culex pipiens Mosquitoes by Wolbachia Influences Cytoplasmic Incompatibility
Source: PLoS Pathog. 2013 Oct 31;9(10):e1003647. doi: 10.1371/journal.ppat.1003647 (PMC3814344; doi:10.1371/journal.ppat.1003647)
Supplement: Table S5 — List of oligonucleotides used. (DOC) [file ppat.1003647.s006.doc]

Table S5.

| CPIJ015950 / CPIJ005623 discrimination PCR primers | |
| --- | --- |
| 56ntindel-F | GTGTCCAACGCCCATCTC |
| 56ntindel-R | AACGTGCTGTTCGCCTTC |
| RpS7-F | AAGGCCATCGTCATCTACG |
| RpS7-R | ACTTCTCCATCTCGCATTCC |
| qRT-PCR primers | |
| RPL32fwd | AAGAAGCGCAAGCTGATTGT |
| RPL32rvs | CGACGGGTAATCGAATTTGT |
| CPIJ005623F1 | GCATGCACGTCGAAGAAATA |
| CPIJ005623R1 | GCCGCTTGAACTTCTTCTTG |
| CPIJ005623F3 | TAAAGCCTGCGGGCTGTGCC |
| CPIJ005623R3 | TCCGCGCCCTCGCACAATTT |
| CPIJ005623F6 | AGCTGCGGAAGCACTACAAG |
| CPIJ005623R6 | GCTCAATCATCCAGCACTTTT |
| RpS7fwd | CCTGGAGCTGGAGATGAACT |
| RpS7rvs | ACGATGGCCTTCTTGTTGTT |
| T7-tailed primers for gene knockdown | |
| CPIJ005623-T7F | TACAAGCTGTCGTGCGATGT |
| CPIJ005623-T7R | TGCTTCTTGAGGGAACCTTG |
| LacZ-T7F | AGAATCCGACGGGTTGTTACT |
| LacZ-T7R | CACCACGCTCATCGATAATTT |
| Genotyping primers | |
| WP0348F | AGGAACAATGGCAACAGGAG |
| WP0348R | AAATTCGCTGTGGAATGAGG |
| WP01286F | TGCTATGAGTTGGCAGAACG |
| WP01286R | AATTGCCATTGTCCTCTTCG |
| WP0455F | TCAACCGGTGGAATTGTTAT |
| WP0455R | TGAGGCAAACATTACGTTCA |
| WP01337F | TACATTGTGCGGCAAGAAAA, |
| WP01337R | CAATCCCTAAATGCCCTTCA, |
| molank1fwd | ccattttgttatgcaaagaagcaaatag |
| molank1rvs | attgcagctatgaacgcaactattcc |
| molank2fwd | gctaagttaagtttagaattaataaagtgcctc |
| molank2rvs | CATTGCATTTCTGCCGCTACCAG |
| molank3fwd | attaaagctgccagaaggttttacaatagg |
| molank3rvs | tatgcagcggtgtatcactgcattg |
| LysoMolfwd | Tggtagaaagcaagaggattaaagtgagc |
| LysoMolrvs | cgattccacttacttcatgtggctc |
| MolTPRfwd | Gcagttattatagctgaagtatttgtgatgc |
| MolTPRfwd | Tggataatcagcagcaagtctcagagtt |
| MolTPRrvs | aacttgaccagcagaagtaactcctctc |
| WP0292FWD | gttgcacaccattacacgtagcag |
| WP0292RVS | CAGCGGTGTCTTTCCATATTTGTC |
| WP0297FWD | cgcaaatataaaagagctgagtgc |
| WP0297RVS | ccactaacctcaaaccccttg |
| WP0430p1FWD | gaggtggcatgtgcttctta |
| WP0430p1RVS | cgctgcatcagttcctagat |
| WP0430p2FWD | gctggtaatggtaacgcaag |
| WP0430p2RVS | gtgccaaagcatcagattca |
| WP0512FWD | actcgattctgaggaagcaga |
| WP0512RVS | cggtaagctggccatattcat |
| WP0599P1FWD | cctgaaaccggagaaatcaa |
| WP0599p1RVS | ACGAGTAAATGGTGCATGGA |
| WP0599P2FWD | AGCATCACCTGTGACTGGTTT |
| WP0599p2RVS | GGCTACTCATGTACCGGACAG |
| WP0666FWD | AATAGCAGGGATTGCTGGTG |
| WP0666RVS | AAGTTGAGCTTGTTCAGGAAGG |
| WP0713FWD | GAGCGTCTTACACTTTCTCCTGA |
| WP0713RVS | caccagatcttattctacgatgtcc |
| WP0717FWD | TCAATTGGGCAAAGGTGACT |
| WP0717RVS | agacttgagcggaccaacat |
| WP0752FWD | gcttaacatcagcagttctatttc |
| WP0752RVS | agttcgtcatcaccctttttg |
| WP0753FWD | catccaaatctggaaaaaga |
| WP0753RVS | tagcctgaattcctaagatctatgg |
| WP0848FWD | aaggttctcatggaatgagaatg |
| WP0848RVS | tcccgttccaccaaaaataa |
| WP0935FWD | gagcgtcacttctggctttc |
| WP0935RVS | tgtatcatctgtccacgcaag |
| WP01050FWD | GCAGCCAAGGAGTTTTATGAG |
| WP01050RVS | GAGCGGATTTTTATCCTCCA |
| WP01336FWD | tggacataaagttgctgagga |
| WP01336RVS | cattgcttcacgctctgttg |
| Cloning primers | |
| WD062FWD | gcatcccttttGGTACCctttatttggtag |
| WD0626RVS | CTTTCGAGGAGCTCCAAATCAATTTCTC |
| Wd0508FWD | gtaaaggTACCgagttcagtttgtcttg |
| Wd0508RVS | TAGGAGCTCAGTGAAGTCAAATCAATTTTTC |
| MolTRFWD | CTGTGCGGCCGCTATGGCAAATATC |
| MolTRRVS | gagctctttgttgaatttcgcctaaag |
| WSPFWD | TTAACTTTAGTTGTTACTTAGGGTACCAAAGTGG |
| WSPRVS | ACTGAGAGCGGCCGCAAATTAAACGCTACTCC |
